# Volition and the Brain – Revisiting a Classic Experimental Study

**DOI:** 10.1016/j.tins.2018.04.009

**Published:** 2018-07

**Authors:** Chris D. Frith, Patrick Haggard

**Affiliations:** 1 Institute of Philosophy, University of London, Senate House, Malet Street, London WC1E 7HU, UK; 2 Wellcome Centre for Human Neuroimaging at University College London, 12 Queen Square, London WC1N 3BG, UK; 3 Institute of Cognitive Neuroscience at University College London, 17 Queen Square, London WC1N 3AR, UK; 4 Laboratoire de Neurosciences Cognitives, Département d’Études Cognitives, École Normale Supérieure, 29 Rue d’Ulm, Paris 75005, France

**Keywords:** Libet, volition, agency, responsibility, regret, free will

## Abstract

In 1983 Libet *et al*. demonstrated that brain activity associated with a voluntary act precedes conscious experience of the intention to act by several hundred milliseconds. The implication that it is the brain, rather than ‘free will’, that initiates voluntary acts has been discussed ever since by philosophers and lawyers, as well as by scientists. We show here how Libet’s original study gave rise to an entire research field of experimental investigations of volition.

## What Was Done in the Study?

The 1983 study by Libet *et al*. [1] investigated the brain processes underlying the awareness of intending and initiating voluntary, endogenous actions. Participants were asked to make a simple manual movement at a time of their own choice. Neural activity preceding the initiation of action was recorded by averaging electroencephalography (EEG) traces over several trials to produce an event-related potential (ERP). In addition, Libet and colleagues asked their participants to report the time at which they ‘first felt the urge to act’. The participants noted and reported the moment at which they experienced the subjective ‘urge to act’ by looking at an oscilloscope spot rotating like a clock hand. The study thus continued a long tradition of ‘mental chronometry’ – investigating the content of subjective experience by measuring the timing of experience [2]. This surprisingly simple method yielded important insights into the temporal relations between an action, the neural processes that precede it, and the subjective experience of initiating it. Libet *et al*. found a neural precursor of voluntary action, namely the ‘readiness potential’ (RP), which began on average 635 ms (but with a range from −1200 to −225 ms) before action. Nevertheless, the subjective experience of willing the action occurred only some 200 ms before the action. Because causes must precede effects, Libet *et al*. argued that this temporal order rules out the possibility that consciously willing the action caused the RP and the initiation of action. This leaves open the possibility that the RP causes the subjective experience of will (but see [3]).

## Why Does It Matter?

Libet’s conclusion may seem unsurprising to the modern materialist neuroscientist, who views conscious experience as a consequence of brain activity and not as some extrinsic cause of brain activity. The result has been replicated, with several similar studies reporting similar values for the time of conscious intention. Nevertheless, the result contrasts dramatically with the notion of voluntary action that dominates in folk psychology, in modern western culture [4], in philosophy, and in the law [5]. Indeed, the legal definition of action involves both a physical movement event *(actus reus)* and a conscious intent *(mens rea)* that stands in appropriate causal relation to the act. Conscious intention is considered to be a prerequisite for voluntary action, and thus for responsibility. Libet *et al*. claimed to demonstrate that voluntary actions are, in fact, initiated unconsciously. This view has profound implications for philosophical, political, and legal theories of individual autonomy and consciousness. The paper provides a striking example of the impact of neuroscience on concepts of human nature. Perhaps unsurprisingly, both the methods and interpretation of the study have been hotly debated, but, despite its shortcomings, the work has left a long-lasting mark on these debates, and has been referred to frequently in the decades since its publication.

## The Importance of the Outcome of an Action

A frequent criticism of Libet’s paradigm has been that the action involved – lifting a finger – is relatively trivial. This is an example of an action without any substantial consequences. Outside laboratory settings, most actions are undertaken so as to produce outcomes. Actions are the only means by which we can influence the world and the people in it. It is this aspect of action that makes volition important. The phenomenology of volition has been characterised as ‘thin and elusive’, but we have a vivid sense of agency – the experience of controlling the world through our actions.

Follow-up studies have used the same techniques to investigate the experience of actions and their outcomes. This work discovered a phenomenon of intentional binding in subjective experience. The time between an action and its outcome is perceived as being shorter than the objective time interval. When we intentionally press a button to cause a sound some 250 ms later, the interval seems to be shorter than when our finger is passively moved by an external force to press the button. This binding experience depends on the extent to which we believe that the outcome is being caused by our action [6]. The more we care about the outcome the greater the binding and the associated sense of agency. Further, interestingly, binding increases for decisions with a moral rather than an economic component [7]. The different components of agency – the intention and its outcome – can be linked with specific brain regions. The pre-supplementary motor area (pre-SMA) is concerned with the representation of the intention to make a specific movement, while inferior parietal cortex is concerned with a predictive internal model of the upcoming movement. Damage to both pre-SMA and inferior parietal cortex can alter the experience of agency [8].

Libet’s original paradigm also neglected the process of choosing between alternative possible actions. Most real-life situations involve a decision between two or more different actions with real consequences. Before choosing which action to initiate we should reflect on which outcome we want to achieve and which action will best achieve that outcome. Once the action has finally been chosen it can then be initiated. There is evidence that the time of ‘the urge to move’ is associated with the point in this decision-making process at which the action has been chosen. For example, when the choice is between using the left or the right hand, the time of the ‘urge’ relates to the time of the emergence of the lateralised readiness potential, the neural signal which reflects which hand is about to be used ([3], but see [9]).

But what is happening before this final choice is made? Matsuhashi and Hallett [10] proposed that people may be latently aware of an intention to move. They may be thinking about moving, but lack the higher level meta-conscious awareness that this is what they are thinking about. Such meta-conscious awareness may be necessary to make a report. Latent awareness can be demonstrated by delivering an interrupting probe signal before becoming fully aware. Participants were asked to suppress their action if they were thinking about moving when they heard the probe (a randomly occurring tone). The distribution of tone times relative to actions suggested that people start thinking about moving about 1.4 s before the movement occurs. This is well before the time of the ‘urge to move’, in Libet’s original data, although still slightly later than the typical onset of the readiness potential.

This period, during which people are choosing which response to make, is associated with activity in dorsolateral prefrontal cortex (dlPFC) [11]. However, to be clear – this does not imply that this brain region is the origin of free will. dlPFC is part of the outermost of a series of action loops involving the basal ganglia, enabling choices to be made in the widest possible context.

## Regret and Responsibility

Our recollection of thinking about which action to choose can give rise to regret – the feeling ‘I wish I had chosen otherwise’ when an unsatisfactory outcome is revealed. The feeling of regret emerges relatively late in human development (∼9 years of age), and is impaired after frontal lobe lesions [12]. The feeling of regret is closely related to the feeling of responsibility – I could have chosen otherwise and therefore I am responsible for making a bad choice.

Perhaps the belief that we could have chosen otherwise is an illusion. Nevertheless, assignment of responsibility is an important aspect of human society, being crucial for the allocation of blame and punishment. For example, in law, children under a specified age are not considered to be responsible for their actions, presumably because they are not considered to have sufficient capacity for rational deliberation about their actions. People may also be judged not to be responsible for their actions in situations where they act while they are unconscious [4] – for instance during some types of epileptic seizures or while sleep-walking. In these cases, an absence of consciousness is taken to imply a lack of control. In law, this relation is used to justify the defence of automatism [13].

The belief that people are responsible for their actions has an important role in maintaining social cohesion. Unless we punish those among us who flout norms of cooperation the advantages accruing from group living and mutual cooperation could dissipate [14]. However, we only punish individuals who we consider to be responsible for their actions, and we recognise exemptions, including children and some patients with mental health conditions.

## What Role for Conscious Intention?

The actions associated with regret and responsibility are typically pre-planned. In their paper Libet *et al*. reported that, although some actions involved ‘pre-planning’, others were experienced as ‘spontaneous’ and ‘freely capricious in origin’. This distinction seems to have been both part of Libet’s instructions to participants and also part of the reports by the participants, and its significance for interpreting the data is therefore unclear. Endogenous actions can be pre-planned and deliberate to a greater or a lesser degree. RPs before pre-planned actions, which Libet termed ‘type I’, had earlier onsets and larger amplitudes than RPs preceding more spontaneous actions, termed ‘type II’. This distinction is made nowhere else in the literature, as far as we know, and seems to be have been largely forgotten or ignored by Libet’s readers. Nevertheless, both forms of RP began before conscious intention, supporting Libet’s argument about temporal precedence.

Most subsequent work on volition has focussed on Libet’s ‘type I’ actions in which pre-planning is involved because participants are required to choose between actions with different outcomes.

Libet *et al*. chose to focus on more spontaneous voluntary actions (type II) that presumably have greater ‘randomness’. This links to another line of research using tasks in which participants are explicitly asked to respond at random (e.g., [12]). Interestingly, however, it is fairly difficult for humans to behave randomly. One strategy that might overcome this problem is to rely on stochastic noise, which is present in the brain, as in all biological systems. Schurger and colleagues [15], for instance, suggest that, given a weak imperative to move, the precise moment at which the decision threshold for movement is crossed is largely determined by spontaneous fluctuations in neuronal activity. Is it possible that people can somehow take account of such fluctuations and use them to generate ‘spontaneous’ actions? In this case, the observation that brain activity precedes the conscious ‘urge to act’ would no longer be inconsistent with our beliefs about the nature of volition.
